# H_2_S Prodrug, SG-1002, Protects against Myocardial Oxidative Damage and Hypertrophy In Vitro via Induction of Cystathionine β-Synthase and Antioxidant Proteins

**DOI:** 10.3390/biomedicines11020612

**Published:** 2023-02-18

**Authors:** Rahib K. Islam, Erinn Donnelly, Erminia Donnarumma, Fokhrul Hossain, Jason D. Gardner, Kazi N. Islam

**Affiliations:** 1 Departments of Pharmacology and Experimental Therapeutics, Genetics, and Physiology, Louisiana State University Health Sciences Center, 1901 Perdido St., New Orleans, LA 70112, USA; rislam@lsuhsc.edu (R.K.I.); donn7171@vandals.uidaho.edu (E.D.); fhossa@lsuhsc.edu (F.H.); jgardn@lsuhsc.edu (J.D.G.); 2 Mitochondrial Biology Group, Institute Pasteur, CNRS UMR 3691, 75015 Paris, France; erminia.donnarumma@pasteur.fr; 3 Agricultural Research Development Program, College of Engineering, Science, Technology and Agriculture, Central State University, 1400 Brush Row Road, Wilberforce, OH 45384, USA

**Keywords:** H_2_S, SG-1002, oxidative stress, reactive oxygen species (ROS), cardiovascular diseases, gasotransmitters, antioxidants, cardiomyocytes

## Abstract

Endogenously produced hydrogen sulfide (H_2_S) is critical for cardiovascular homeostasis. Therapeutic strategies aimed at increasing H_2_S levels have proven cardioprotective in models of acute myocardial infarction (MI) and heart failure (HF). The present study was undertaken to investigate the effects of a novel H_2_S prodrug, SG-1002, on stress induced hypertrophic signaling in murine HL-1 cardiac muscle cells. Treatment of HL-1 cells with SG-1002 under serum starvation without or with H_2_O_2_ increased the levels of H_2_S, H_2_S producing enzyme, and cystathionine β-synthase (CBS), as well as antioxidant protein levels, such as super oxide dismutase1 (SOD1) and catalase, and additionally decreased oxidative stress. SG-1002 also decreased the expression of hypertrophic/HF protein markers such as atrial natriuretic peptide (ANP), brain natriuretic peptide (BNP), galectin-3, TIMP1, collagen type III, and TGF-β1 in stressed HL-1 cells. Treatment with SG-1002 caused a significant induction of cell viability and a marked reduction of cellular cytotoxicity in HL-1 cells under serum starvation incubated without or with H_2_O_2_. Experimental results of this study suggest that SG-1002 attenuates myocardial cellular oxidative damage and/or hypertrophic signaling via increasing H_2_S levels or H_2_S producing enzymes, CBS, and antioxidant proteins.

## 1. Introduction 

Sulfur has a critical role in protein structure/function and redox status/signaling in all living organisms. Although a sulfur containing molecule, hydrogen sulfide (H_2_S), is now recognized as a central player in physiology and pathobiology, the full scope and depth of sulfur metabolome’s impact on human health and longevity has been vastly underestimated and is only starting to be grasped. Since many pathological conditions have been related to abnormally low levels of H_2_S in blood and/or tissues, and are amenable to treatment by H_2_S supplementation, development of safe and efficacious H_2_S donors deserves to be undertaken with a sense of urgency; these prodrugs also hold the promise of becoming widely used for disease prevention and as antiaging agents [1]. One such prodrug is an SG-1002, a precursor to a natural-occurring molecule, H_2_S, for which deficits have been shown to exist in a number of serious diseases including cardiovascular disease (CVD), type II diabetes, cancer, hypertension, etc. [1]. 

DATS (diallyl trisulfide), DBTS (dibutenyl trisulfide), TC-2153 (benzopentathiepin 8-trifluoromethylv-1,2,3,4,5-benzopentathiepin-6-amine hydrochloride), and SG-1002 have the potential as pharmacological therapeutic agents that collectively could prove to be useful in the treatment myriad of disease conditions related to oxidative stress and cellular damage inflicted by reactive oxygen species (ROS) including most aging related diseases [1]. In contrast to other donors, SG-1002 is an H_2_S prodrug and only operates through H_2_S signaling. Other donors elicit pharmacologic effects that are partially through H_2_S. 

Among the known H_2_S prodrugs, SG-1002 is unique in that it lacks a carbon-based scaffold. The absence of a carbon-based scaffold has various advantages for SG-1002 as a medicinal agent. As the number of atoms in the scaffold increases, a given dose contains decreasing amounts of pharmacophore; further, toxicity is frequently scaffold dependent. Additionally, it is not a therapeutic targeted-based agent, it disobeys drug likeness laws, it has a 100% prodrug-to-H2S conversion efficiency, and it is bioactivated. SG-1002 is an α-sulfur rich microcrystalline material that is water insoluble and has traces of ionic chemicals (sodium sulfate, sodium thiosulfate, and sodium polythionates) that have a substantial influence on its physicochemical behavior [1]. 

Furthermore, SG-1002 is orally active, which is a great benefit as a medicinal substance. The oral route of administration increases patient compliance because it avoids more intrusive methods like injections and/or infusions, which often require patients to visit hospitals or health facilities. Additionally, SG-1002 has entered clinical studies where safety and early efficacy data for two distinct purposes have been established. It is also effective in numerous disease models. 

To the best of our knowledge only in one case (SG-1002) has safety been demonstrated in a formal Phase 1 clinical study [2], so to realize the therapeutic potential of these four agents, it will be necessary to invest considerable resources to carry out the required clinical trials. Encouraging results in animal models have been obtained with SG-1002 in heart failure (HF), atherosclerosis, ischemic damage, and Duchenne muscular dystrophy [1]. Based on previously published articles on the roles of SG-1002 in several disease models, the present study was undertaken to determine the effects of SG-1002 on oxidative stress induced hypertrophic signaling in HL-1 cells. The HL-1 cell line is derived from an AT-1 mouse atrial cardiomyocyte tumor and has several key advantages over other types of cardiac cells. It can be recovered from frozen and passaged indefinitely while still maintaining its differentiated biochemical and morphological features, as well as the ability to contract. Because of these properties, HL-1 cardiomyocytes can be used to model the effects of HF, hypertrophy and oxidative stress in vitro [3].

Once thought of only as a toxic gas, H_2_S now belongs to a class of compounds known as gasotransmitters. H_2_S along with carbon monoxide (CO) and nitric oxide (NO) are endogenously produced gases that are required for cardiovascular homeostasis [4]. Previous studies have shown that NO is highly cytoprotective and that maintaining NO bioavailability is protective against the development and progression of HF [5,6,7]. Similarly, to H_2_S, NO levels are reduced in HF patients compared with healthy controls [5]. Endothelial nitric oxide synthase (eNOS) is elevated in the presence of H_2_S leading to the increased levels of circulating NO [4,8]. Similarly, NO therapy was found to increase the levels of H_2_S in murine HF model [9]. Endogenously produced H_2_S exerts a variety of cytoprotective actions in vivo, by acting as an antioxidant and promoting Nrf2 and NRF-1 signaling [10,11,12], and thereby augmenting NO-mediated signaling [4]. 

H_2_S is of particular interest as a possible agent to combat HF because of its ability to promote vasodilation and its anti-inflammatory [13], antioxidant [14] and anti-apoptotic [15] properties. Previous studies have shown that genetic overexpression of the H_2_S producing enzyme cystathionine γ-lyase (CSE) protects against HF while deficiency exacerbates it [8]. H_2_S is a potent antioxidant that can eliminate free radicals and prevent new reactive oxygen species (ROS) from forming, which are particularly detrimental in myocardial infarction/reperfusion (MI/R) injuries [14]. Additionally, H_2_S has recently been implicated as a potential treatment against hypertrophic signaling [16], which is responsible for the pathological remodeling associated with HF. 

Three enzymes are responsible for the endogenous production of H_2_S: cystathionine β-synthase (CBS), CSE, and 3-mercaptopyruvate sulfur transferase (3-MST). H_2_S (and its metabolites) are found in most organ systems, including the heart, liver, kidney, brain, nervous system, lung, airway tissues, gastrointestinal tract, reproductive organs, skeletal muscle, pancreas, synovial joints, connective tissue, cochlea, and adipose tissues [17]. Impaired H_2_S generation as a result of CSE dysfunction has been cited as a significant contributor to pathology in numerous disease states [4,18,19]. Furthermore, H_2_S is critically important to maintaining homeostasis in the cardiovascular system, both in the heart and in the circulation [20,21]. In preclinical models, H_2_S therapy attenuates disease severity by antioxidant activity, promoting angiogenesis, modulating mitochondrial function, reducing inflammation, upregulating antioxidant gene programs, inhibiting cell death, and attenuating fibrosis [8,20,22,23]. 

In mammals, CSE is predominately responsible for the manufacture of H_2_S in the cardiovascular system, while CBS is present in greater quantities in the central nervous system [24]. 3-MST is responsible for manufacturing 90% of the H_2_S in the brain [24]. A study by Islam et al. has shown that NO therapy protects cardiac function in the myocardial ischemia (MI) murine model via induction of H_2_S/H_2_S producing enzymes (CSE and CBS) and antioxidant levels [9]. Through the use of an exogenous H_2_S donor, SG-1002, we attempted to identify a mechanism for the decrease in stress-induced cardiomyocyte hypertrophic signaling. Furthermore, we also sought to ascertain whether this prodrug may promote expression of CBS, CSE, and 3-MST as well as antioxidant proteins, enabling cells to create more endogenous H_2_S. This is the first report demonstrating the beneficial effects of SG-1002 on stress/hypertrophic murine cardiomyocytes.

## 2. Materials and Methods

### 2.1. Cell Culture 

Murine HL-1 cardiac muscle cells were maintained in 10% fetal bovine serum (FBS) containing Claycomb media followed by serum starvation (1% FBS) media for 24 h. Serum starved/stressed cells were treated for 1 h with either 10 μM SG-1002 (Received from Dr. David J. Lefer, LSU, New Orleans), or H_2_O_2_ (500 μM), or hydroxylamine (HA) 10 (μM), or a hypertrophic agonist such as endothelin-1 (ET-1) (10 nM), or in combination. Treated cells were analyzed for mRNA levels (by RTqRT-PCR), H_2_S levels, oxidative stress, and related assays. 

### 2.2. Real Time Quantitative Reverse Transcription Polymerase Chain Reaction (RTqRT-PCR)

RNA was isolated from the stressed HL-1 cells treated without or with 10 μM of SG-1002, or H_2_O_2_ (500 μM) or HA (10 μM) or in combination for 1 h in 1% serum (FBS) containing media. 1 μg of RNA was used for the synthesis of cDNA. TaqMan primers from Life Technology Co, Carlsbad, CA, USA. for ANP, BNP, TGF β1, collagen type III, galectin 3, TIMP1, SOD-1, catalase, CSE, CBS, and 3-MST were used to amplify q-PCR. 18s rRNA was used as a house keeping gene. 

### 2.3. Measurement of H_2_S 

Stressed HL-1 cells treated without or with 10 μM of SG-1002, or H_2_O_2_ (500 μM) or in combination for 1 h in 1% serum (FBS) containing media. The levels of H_2_S were determined in culture media samples by using gas chromatography chemiluminescence method as described previously [9]. 

### 2.4. Immunoblot Assay

The polyclonal antibody for SOD1, catalase, and the monoclonal antibody for GAPDH were purchased from Santa Cruz Biotechnology, Inc., Santa Cruz, CA. Murine HL-1 cardiac muscle cells were maintained in 10% fetal bovine serum (FBS) containing Claycomb media followed by serum starvation (1% FBS) media for 24 h. Serum starved/stressed cells were treated for 1 h with either 10 μM SG-1002. Serum starved/stressed HL-1 cells were treated with or without 10 μM of SG-1002 for 1 h in 1% serum containing media followed protein extraction. The level of SOD1, catalase and GAPDH were determined by using immunoblot analysis [10]. Total protein samples were prepared from treated HL-1 cardiomyocytes using RIPA lysis buffer and quantified using BCA protein assay kits from Pierce, Inc. Waltham, MA, USA. 15 μg of protein samples were loaded in each well and separated were then separated via electrophoresis on a 4–20% Mini-PROTEAN TGX Precast Gel (Bio-Rad Laboratories, Inc., Hercules, CA, USA) and transferred to a 0.45 μM nitrocellulose membrane (Bio-Rad Laboratories, Inc.). Membranes were blocked for at least two hours with Odyssey Blocking Buffer (Li-Cor, Lincoln, NE, USA), diluted 1:1 with PBS followed by probing with primary (such as SOD1, Catalase, and GAPDH) antibodies for 3 h at 4° and fluorescence conjugated secondary antibodies secondary antibodies for 1.5 h at room temperature. Proteins bands were visualized and scanned/quantified by Li-Cor/Odyssey infrared imaging system. GAPDH was employed as a loading control.

### 2.5. Measurement of Advanced Oxidative Protein Products (AOPP)

All reagents or chemicals used in our experiments were purchased from Sigma-Aldrich. Advanced oxidative protein products AOPP Assay Kit was obtained from Abcam (ab242295). AOPP is a simple, reproducible, and consistent system for the detection of advanced oxidation protein products in plasma, lysates, and tissue homogenates. This kit includes a chloramine standard and an AOPP Human Serum Albumin conjugate for use as a positive control. Oxidative stress was measured by determining the levels of AOPP in cultured HL-1 cells after incubation without or with either 10 μM of SG-1002 or 500 μM of H_2_O_2_ or in combination in 1% FBS containing media. AOPP was determined using a spectrophotometric method. Samples were incubated with glacial acetic acid and the absorbance was read at 340 nm. Chloramine T with potassium iodide was used as calibrator. 

### 2.6. Cell Proliferation Assay

Cell viability was determined utilizing a Vybrant^®^ MTT (3-(4,5-Dimethylthiazol-2-Yl)-2,5-Diphenyltetrazolium Bromide) cell proliferation assay kit (Thermo Fisher Scientific, Waltham, MA, USA). Cells were maintained on a 96-well tissue culture plate (Falcon, Corning, NY, USA) in 10% serum and cultured for 24 h in serum starvation (1%) prior to treatment. Cells were then pretreated with DMSO or SG-1002 for 30 min, followed by treatment without or with 10 μM SG-1002 or 500 μM of H_2_O_2_, or in combination for one hour. 10 μL of the 12 mM MTT stock solution was loaded into each well and the plate was incubated in the dark at 37 °C for 2 h. Following the incubation period, 175 μL of the media was removed, and 50 μL of DMSO was added to the remaining 25 μL in each well. The plate was then incubated at 37 °C for 10 min and the optical density was determined using a spectrophotometer at 540 nm. 

### 2.7. Lactate Dehydrogenase (LDH) Cytotoxicity Assay

Stressed HL-1 cells were incubated with either 10 μM of SG-1002, or H_2_O_2_ (500 μM) and or in combination for 1 h in 1% serum containing media. Treated HL-1 cellular cytotoxicity was determined by using the assay kits obtained from Thermo Fisher Scientific.

### 2.8. Measurement of ROS

Cellular ROS were measured using a cellular ROS/superoxide detection assay kit (Abcam, Cambridge, UK). Cells were maintained on a 96-well tissue culture plate (Falcon, Corning, NY, USA) in 10% FBS and cultured for 24 h in serum starvation (1% FBS) prior to treatment. Cells were then pretreated with DMSO or SG-1002 for 30 min, followed by treatment with 500 μM of H_2_O_2_, and/or 10 μM SG-1002 or in combination for one hour in combination in 1% serum containing media. After treatment, cells were loaded with 100 μL/well of ROS superoxide detection solution and incubated for one hour in the dark at 37 °C. The plate was then read from the bottom at excitation 485/20 and emission 528/20. 

### 2.9. Statistical Analysis 

All data in this study were expressed as the mean ± SE from at least three independent experiments from given n sizes. Statistical significance between two groups was determined using the two-tailed Student’s t test. Statistical significance of multiple treatments was determined by one-way analysis of variance followed by the Bonferroni post hoc test when appropriate. A *p* value of <0.05 was considered significant. 

## 3. Results

### 3.1. Time Course and Dose Responses of SG-1002 on H_2_S Production

**Figure 1:**
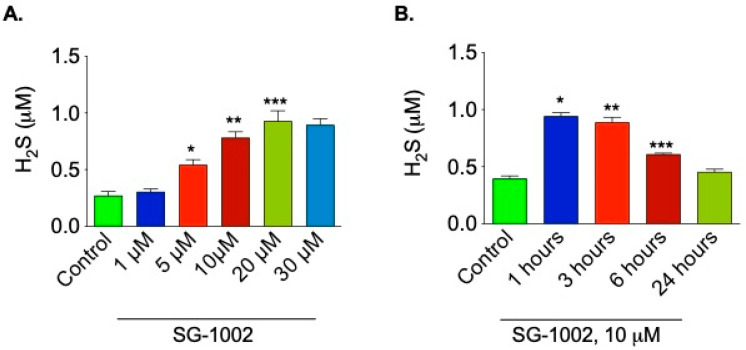
Time course and dose responses of SG-1002 on H_2_S production. HL-1 cardiac muscle cells were incubated either with different concentrations of SG-1002 for 1 h (**A**) or with 10 mM of SG-1002 for several time periods (**B**) in 1% serum containing media followed by the measurement of H_2_S levels in culture media. *, **, and ***. *p* < 0.05 versus (vs.) control (n = 4).

HL-1 cardiac muscle cells were incubated either with different concentrations of SG-1002 for 1 h (Figure 1A) or with 10 μM of SG-1002 for several time points (Figure 1B) in 1% serum containing media followed by the measurement of H_2_S levels in culture media. As can be seen 10 and 20 μM SG-1002 was able to significantly increase H_2_S levels in HL-1 cells (Figure 1A). It was also observed that treatment with 10 μM of SG-1002 produced more H_2_S in 1 h as compared with other time points. This result suggests that SG-1002 induces the production of H_2_S.

### 3.2. Induction of CBS by SG-1002 in Cultured HL-1 Cardiac Muscle Cells 

**Figure 2:**
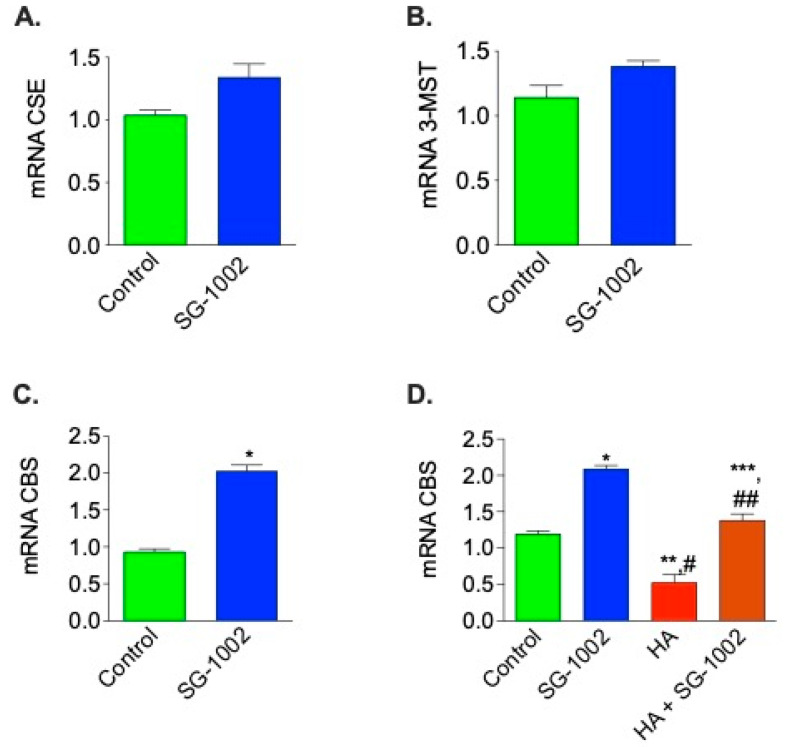
Effects of SG-1002 on mRNA Expression of CBS, CSE, and 3-MST in HL-1 cells. HL-1 cells were incubated with specific reagents as described in Materials and Methods for 1 h in 1% serum containing media. cDNA was prepared from RNA obtained from treated HL-1 cells followed by analysis of mRNA of CSE (**A**), 3-MST (**B**), and CBS (**C**,**D**) using TaqMan PCR assay system. *, *p* < 0.05 vs. control, (**A**–**C**), (*t* test) (n = 4); *, and **, *p* < 0.05 vs. control; #, *p* < 0.05 SG-1002 vs. HA; ##, *p* < 0.05 HA vs. SG-1002 +HA; ***, *p* < 0.05 SG-1002 vs. SG 1002 + HA (D) (Anova) (n = 3).

Treatment with SG-1002 significantly increased (*p* < 0.05) the mRNA expression of CBS in HL-1 cardiac muscle cells cultured for 1 h in 1% serum, as compared to the control. mRNA expression of CSE and 3-MST were also slightly up-regulated compared to the control, though not significantly (Figure 2A–C). We were also interested in whether SG-1002 can induce CBS expression in the presence of an inhibitor of CBS, such as hydroxylamine (HA). We observed that SG-1002 induces CBS mRNA expression even in the presence of HA as compared with HA alone (Figure 2D).

### 3.3. Inhibition of Oxidative Stress by SG-1002 in Cultured HL-1 Cardiac Muscle Cells

**Figure 3:**
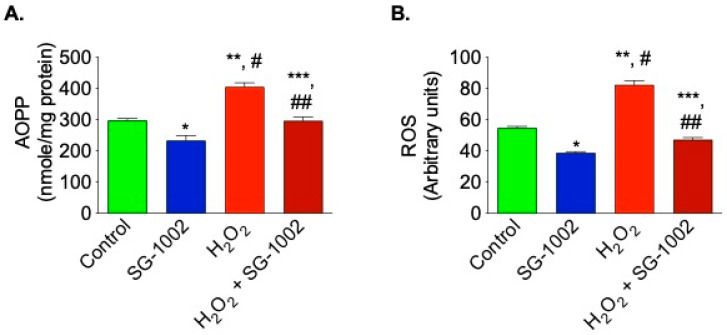
Reduction of oxidative stress in HL-1 cells by SG-1002. Oxidative stress was measured by determining the levels of AOPP (**A**) and ROS (**B**) in cultured HL-1 cells after tread with specific reagents as described in Materials and Methods. The experiment was repeated at least three times to verify the reproducibility. *, and **, *p* < 0.05 vs. control; #, *p* < 0.05 SG-1002 vs. H_2_O_2_; ##, *p* < 0.05 H_2_O_2_ vs. SG-1002 + H_2_O_2_; ***, *p* < 0.05 SG-1002 vs. SG-1002 + H_2_O_2_ (Anova) (n = 4).

Hl-1 cells treated with SG-1002 had significantly lower levels of oxidative stress when compared to the controls. AOPP and ROS levels were measured in 4 treated groups of HL-1 cells following 24 h of serum starvation: DMSO vehicle control, 10 μM SG-1002, DMSO and 500 μM H_2_O_2_ (to further stress the cells), or SG-1002 and 500 μM H_2_O_2_. The cells treated with SG-1002 alone had the lowest levels of oxidative stress (AOPP and ROS), followed by the DMSO-treated control group (Figure 3A,B). The H_2_O_2_ group had the highest levels of oxidative stress and SG-1002 was able to antagonize the effects of H_2_O_2_ (Figure 3A,B).

### 3.4. Effects of SG-1002 on SOD1 and Catalase Levels in HL-1 Cells

**Figure 4:**
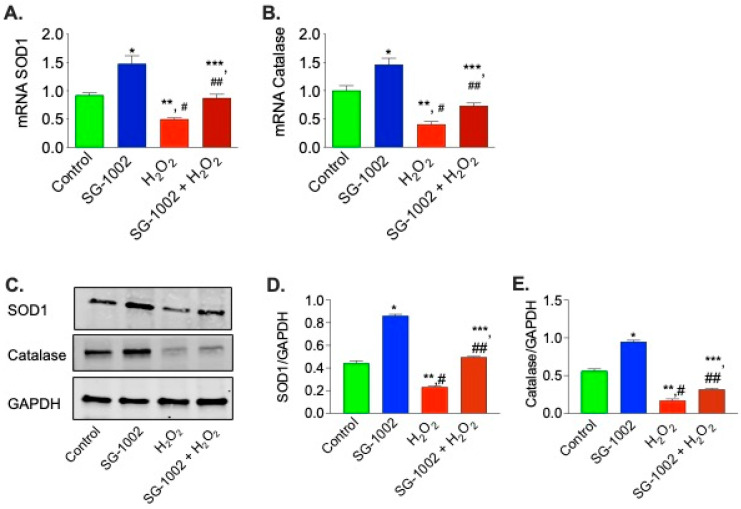
Effects of SG-1002 on mRNA and proteins levels of SOD1 and catalase in HL-1 cells. cDNA was prepared from RNA obtained from treated HL-1 cells followed by analysis of mRNA of SOD1 (**A**) and catalase (**B**) using TaqMan PCR assay system. SOD1 and catalase levels were determined in the protein extracts obtained from treated HL-1 cells by using immunoblot analysis. (**C**) represents blots for SOD1, catalase and GAPDH, (**D**,**E**) represent the quantitation of blots in (**C**). * and **, *p* < 0.05 vs. control; #, *p* < 0.05 SG-1002 vs. H_2_O_2_; ##, *p* < 0.05 H_2_O_2_ vs. SG-1002 + H_2_O_2_; ***, *p* < 0.05 SG 1002 vs. SG 1002 + H_2_O_2_ (Anova), (n = 4).

It was then of interest to determine the levels of antioxidative enzyme genes expression in HL-1 cells were treated without or with either 10 μM of SG-1002, or H_2_O_2_ (500 μM) or in combination for 1 h in 1% serum containing media. cDNA was prepared from RNA obtained from cultured HL-1 cells followed by analysis of mRNA of SOD1 (Figure 4A) and catalase (Figure 4B) using the TaqMan PCR assay system. Treatment of HL-1 cells with SG-1002 significantly increased mRNA expression of SOD1 and catalase. Furthermore, SG-1002 also increased the expression of both enzymes even under oxidative stress induced by H_2_O_2_ (Figure 4A,B). 

Then we were interested in determining the effects of SG-1002 on the protein levels of SOD1 and catalase by utilizing immunoblot analysis in stressed HL1 cells. We found that in stressed HL1 cells, SG-1002 significantly increased SOD1 and catalase levels as compared with the control (Figure 4C–E). This data suggests that SG-1002 protects HL-1 cells from oxidative damage via inducing antioxidant proteins.

### 3.5. Effects of SG-1002 on H_2_S Production and CBS mRNA Expression When Cells Were Cultured under Stress

**Figure 5:**
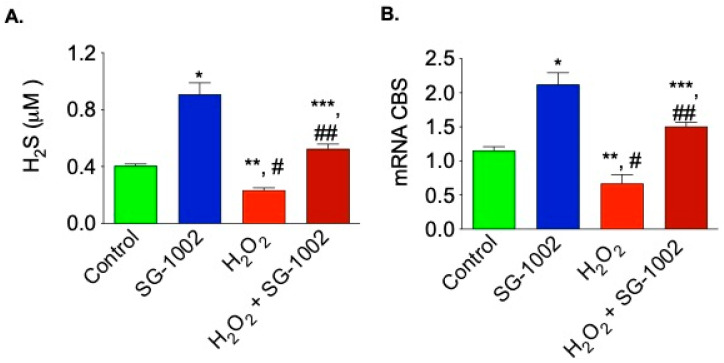
Effects of SG-1002 on H_2_S production and CBS mRNA expression when cells were cultured under stress. HL-1 cells were incubated with specific reagents as described in Materials and Methods for 1 h in 1% serum containing media followed by the measurement of H_2_S levels in culture media (**A**) and analysis of CBS mRNA (**B**) using the TaqMan PCR assay system. *, and **, *p* < 0.05 vs. control; #, *p* < 0.05 SG-1002 vs. H_2_O_2_; ##, *p* < 0.05 H_2_O_2_ vs. SG-1002 + H_2_O_2_; ***, *p* < 0.05 SG-1002 vs. SG 1002 + H_2_O_2_ (Anova) (n = 3).

It was also of interest to examine whether SG-1002 is able to induce production of H_2_S and H_2_S producing enzyme CBS, when cells were treated with H_2_O_2_. As can be seen here under oxidative stress, SG-1002 also significantly induced the production of H_2_S as well as CBS mRNA in HL-1 cells treated with SG-1002 plus H_2_O_2_ as compared with H_2_O_2_ alone (Figure 5A,B).

### 3.6. Effects of SG -1002 on the Expression of HF Biomarkers in HL-1 Cells 

**Figure 6:**
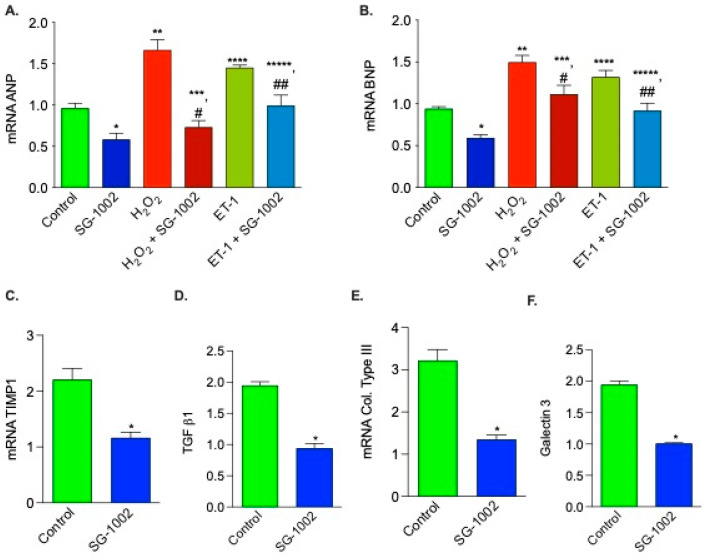
Effects of SG -1002 on the expression of HF markers in HL-1 cells. cDNA was prepared from RNA obtained from treated HL-1 cells as described in Materials and Methods followed by analysis of mRNA of ANP (**A**), BNP (**B**), TIMP1 (**C**), TGF b1 (**D**), collagen (col) type III (**E**), and galectin 3 (**F**) using the TaqMan PCR assay system. *, **, and ****, *p* < 0.05 vs. control; #, *p* < 0.05 SG-1002 vs. H_2_O_2_ + SG-1002; ***, *p* < 0.05 H_2_O_2_ vs. SG-1002 + H_2_O_2_; ##, *p* < 0.05 ET-1 vs. SG-1002 + ET1; *****, *p* < 0.05, SG-1002 vs. ET1 + SG-1002 (Anova) (**A**,**B**) (n = 4); *t* test (**C**–**F**) (n = 3).

It is well known the expression of both ANP and BNP are elevated during hypertrophy or HF. Therefore, it was of interest to determine the levels of these gene expression in H_2_O_2_ and ET-1-treated HL-1 cells. As can be seen in Figure 6, SG-1002 significantly inhibited H_2_O_2_ or ET-1 induced ANP (Figure 6A) and BNP (Figure 6B) in HL-1 cells. 

We also tested the effects of SG-1002 on mRNA expression of other HF biomarkers, for example, TIMP1, TGF b1, collagen type III, and galectin 3. mRNAs of all these upregulated biomarkers in stressed HL1 cells are markedly decreased by SG-1002 (Figure 6C–F). Inhibition of hypertrophic genes expression in stressed HL-1 cells by SG-1002 suggests that SG-1002 plays important roles in the protection of HL-1 cells from oxidative damage.

### 3.7. SG-1002 Decreases Oxidative Stress-Induced Cellular Death and Cytotoxicity in Muscle Cells 

**Figure 7:**
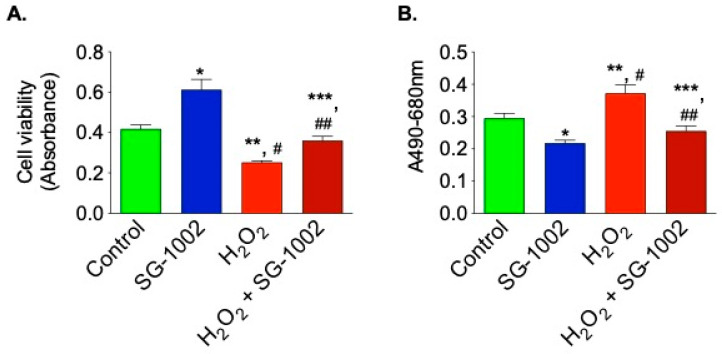
SG-1002 decreases oxidative stress-induced cellular death and cytotoxicity in HL-1 cardiac muscle cells. HL-1 cells were incubated with specific reagents as described in Materials and Methods. Cell viability (**A**) and cytotoxicity (**B**) were determined by using MTT and LDH cytotoxicity assays, respectively. * and **, *p* < 0.05 vs. control; #, *p* < 0.05 SG-1002 vs. H_2_O_2_; ##, *p* < 0.05 H_2_O_2_ vs. SG-1002 + H2O2; ***, *p* < 0.05 SG-1002 vs. SG 1002 + H_2_O_2_ (Anova) (n = 3).

Next, the effects of SG-1002 on cells viability and cytotoxicity were measured using HL1 cells treated without or with either 10 μM of SG-1002, or H_2_O_2_ (500 μM) and or in combination for 1 h in 1% serum containing media. Cell viability (Figure 7A) and cytotoxicity (Figure 7B) were determined by using MTT and LDH cytotoxicity assays, respectively. 

Treatment with SG-1002 alone markedly improves cell viability when compared with controls. Additionally, when the cells were further stressed with 500 μM H_2_O_2_, treatment with SG-1002 was able to significantly (*p* < 0.05) improve cell viability as determined by MTT assay (Figure 7A). Cytotoxicity was measured by using lactate dehydrogenase (LDH) cytotoxicity assay (Figure 7B). Treatment with SG-1002 significantly lowered levels of LDH when compared to controls when HL-1 cells were under oxidative stress (Figure 7B). 

The current data unambiguously show that SG-1002 reduces myocardial cellular oxidative damage and/or hypertrophic signaling by elevating H_2_S levels, CBS, the enzyme that produces H_2_S, and antioxidant proteins.

## 4. Discussion 

DATS, DBTS, and SG-1002 have the potential to be used prophylactically for a variety of purposes, including the promotion of immunity, prevention or slowing the onset of chronic-degenerative diseases, and protecting the vital organs from damage induced by other drugs such as paracetamol, corticosteroids, chemotherapy agents, etc. SG-1002 has an advantage in this context, namely its efficient conversion into H_2_S without causing halitosis and body odor. Novel, long acting, and controllable H_2_S-based therapeutics (i.e., H_2_S donors, prodrugs, and H_2_S enzyme activators) may represent valuable candidates for drug development. However, challenges to their use in clinical practice remain. Currently, H_2_S donors have poor pharmacokinetics with a very short half-life and uncontrolled release [20]. Two commercially available inorganic salts, Na_2_S and NaHS, rapidly increase free H_2_S concentration, but this increase is short-lived (seconds). These compounds also have a narrow therapeutic dose window leading to potential toxicity [25]. Because it is not possible to supplement H_2_S levels in a sustained manner, use of these compounds for treating chronic disorders, such as HF, is not clinically feasible. Additionally, given the toxicity of supraphysiological H_2_S levels, it is critical that novel H_2_S agents with favorable pharmacokinetic profiles are developed specifically for clinical application. Based on the published literature regarding the importance of H_2_S on cardiac homeostasis and the benefit of SG-1002 in several disease models, the present study was undertaken to determine the effects of this prodrug, SG-1002, on oxidative stress induced hypertrophic signaling in stressed murine cardiomyocytes (HL-1 cells). In this study, we investigated a mechanism of the reduction in stress induced cardiomyocyte hypertrophic signaling through the use of an exogenous H_2_S donor, SG-1002. Our aim was to determine whether this prodrug could induce expression of H_2_S producing enzymes (CBS, CSE and 3-MST) as well as antioxidant protection, which would allow cells to produce more endogenous H_2_S. 

Low levels of H_2_S in blood or tissue has been correlated with the onset of disease onset of disease states related to oxidative cell damage, chronic inflammation [26], immune dysfunction [26,27], endoplasmic reticulum (ER) stress [28,29], dysregulation of mitochondrial bioenergetics [30], and hyperproliferation of cells or viruses [31], suggesting causal links that are actively being investigated. Furthermore, an inverse link between illness progression and H_2_S in blood and/or tissues has been established in some cases [32,33,34]. So called ‘‘H_2_S-poor’’ disease states that are amenable to correction by H_2_S donors include [35,36] aging, ischemia, cardiac hypertrophy, HF, liver disease (cirrhosis, steatosis), hypertension, atherosclerosis, endothelial dysfunction, diabetic complications, preeclampsia, Alzheimer’s disease (AD), and Huntington’s disease (HD). 

It has been reported that oxidative stress results in cardiac contractile dysfunction and is a key player in the transition from compensatory cardiac hypertrophy to decompensated HF [37]. H_2_S is known to reduce oxidative stress in two ways: by direct inactivation of oxidant species and via upregulation of endogenous antioxidant defenses [38,39]. In our study we observed treatment of stressed HL-1 cells with H_2_S prodrug, SG-1002, decreased ROS and AOPP (oxidative stress markers) and increased SOD1 and catalase via induction of CBS/H_2_S. SG-1002 may also attenuate oxidative stress via a third mechanism: recoupling of eNOS. Uncoupled eNOS (as seen in HF) results in decreased NO generation, and leads to excessive peroxynitrite concentrations [40].

L-cysteine is converted into H_2_S via CSE. In vivo studies have shown that H_2_S protects against acute MI/R injury [39]. In a recent study, wild-type mice and CSE-knock out (KO) mice underwent transverse aortic constriction (TAC) and then received either SG-1002 or vehicle. CSE-KO mice developed worsened cardiac remodeling and function compared to control mice. Interestingly, CSE-KO mice that received SG-1002 exhibited improved cardiac remodeling and function compared to vehicle treated mice [11]. SG-1002 has also been examined in preclinical studies of HF [8]. SG-1002 attenuates left ventricular (LV) remodeling and dysfunction in a pressure overload model of HF [8]. Administration of SG-1002 significantly decreased disease markers and increased both H_2_S and NO levels [8]. A subsequent phase 1 clinical trial was performed using SG-1002 for a 21-day period in healthy and HF subjects. In this trial it was observed that SG-1002 significantly augments circulating H_2_S levels in HF patients and healthy subjects. SG-1002 was also found to be well tolerated and safe at all doses tested [2]. 

In a model of acute limb ischemia, the proangiogenic effects of SG-1002 were evaluated (ALI). Pigs were given either a placebo or SG-1002 for the 35-day research after they had the intravascular occlusion technique to induce ALI. Moreover, SG-1002 also preserved existing capillaries in ischemic limbs to a 1.6 times greater extent than in pigs that received placebo [41]. 

Terminally ill children between 18 month and 14 years of age presented with osteosarcoma, hydrocephalus with cancerous tumor, medulloblastoma, squamous cell carcinoma, and acute lymphoblastic leukemia (all refractory to chemotherapy and/or radio therapy) were treated at doses between 1200 mg and 3600 mg SG-1002 daily, no adverse reaction was reported. Additionally, improvements in fatigue, inflammation, pain, headache, cardiac function, blood sugar regulation and reduced tumor volume were reported [1]. Bibli et al. found that treatment of human umbilical vein endothelial cells with SG-1002 induced protein S-sulfhydration and protection of membrane lipids from peroxidation [42]; these in vitro findings are consistent with the significant increases in H_2_S levels of blood and tissues attained when SG-1002 is orally administered to mice [8], swine [41], and humans [2].

Another recent study investigated the effects of SG-1002 on homocysteine induced cardiac remodeling and dysfunction. CBS^+/−^ and CBS^+/+^ (WT) mice treated with SG-1002 in their chow. At baseline, CBS^+/−^ mice showed an increased afterload (increased end systolic pressure with conserved stroke volume). This phenotype is abolished with SG-1002 treatment by reducing end systolic pressure and, at the same time, significantly increasing the end-diastolic volume. Additionally SG-1002 was found to prevent the CBS^+/−^ mice from developing pathological cardiac remodeling [43]. This work established that increasing H_2_S levels with SG-1002 in the setting of HF increases cardiac mitochondrial content/function and improves cardiac function via AMPK activation [44]. Another recent study showed the impact of SG-1002 on atherosclerosis in mice. In this study, the left carotid artery was partially ligated. Mice that received SG-1002 had significantly reduced plaque formation, indicating a potential anti-atherosclerotic effect of SG-1002 [45]. 

Many previous studies have established that oxidative stress/ROS exacerbate HF while antioxidants protect against it [46]. Certain ROS, such as hydrogen peroxide (H_2_O_2_), act as signaling molecules [47] for the immune system and helps recruit white blood cells to initiate healing to damaged tissues [48]. They are usually eliminated via interactions with superoxide dismutase, catalase, glutathione peroxidase, and peroxiredoxins [49]. Similarly, in our current study it was observed that overproduction of ROS and AOPP in stressed HL-1 cells were scavenged by catalase and SOD1 induced by SG-1002 treatment. In many chronic conditions, more ROS are produced than can be eliminated, causing an imbalance between the antioxidant and oxidant systems. This imbalance is toxic and causes damage to many organelles including the mitochondria. In the setting of HF, this imbalance can cause changes in normal autophagy pathways [50], worsening arteriosclerosis [51], and persistent low levels of systemic inflammation [52]. 

A study found that mice that had undergone MI/R had smaller infarct sizes and improved LV function when treated with a H_2_S donor [53]. Our previous study in HF murine model has shown that NO mediated induction of H_2_S producing enzymes and/or H_2_S causing the activation of antioxidant gene regulated transcription factor, Nrf2 resulting in increased antioxidant protection of cells from damage [9]. Present study shows that treatment of stressed HL1 cells with a H_2_S donor, SG-1002, increased the levels of the H_2_S-producing enzyme, CBS, as well as antioxidant proteins, SOD1 and catalase, leading to the protection of cells from damage. This suggests that SG-1002 works at least in part by increasing expression of antioxidant systems. H_2_S is a powerful antioxidant compound that is capable of scavenging free radicals in vitro [54]. Similarly, in the present study it was observed that, when stressed cardiomyocytes were treated with SG-1002, they had lower levels of oxidative stress because of the induction of H_2_S/ or H2S producing enzymes such as CBS. We also noted that the cells that were further stressed with H_2_O_2_ and treated with SG-1002 had much lower levels of oxidative stress compared to the H_2_O_2_ alone group, but higher levels than both the SG-1002 alone and control group, suggesting that high levels of pre-existing oxidative stress impair its effect. 

While some studies point to apoptosis after MI/R being cardioprotective, others point to it being a detrimental to LV function. Changes in normal cell death and apoptosis are known to occur in HF [55]. We assessed the effects of SG-1002 on cell death and cytotoxicity in stressed HL-1 cells. The assays we used included MTT and LDH cytotoxicity, both of which provide information about the amount but not the pathways that cause cell death and cytotoxicity. In order to determine which pro-survival pathways are being affected by treatment with SG-1002, future studies may investigate increases in genes and proteins related to improved survival such as beclin-1, or decreases in pro-apoptotic markers, such as bax, caspase-3, or some other related genes. 

Another way sulfide donor is said to have cardioprotective effects is by attenuating hypertrophy. A previous study found that mice treated with SG-1002 had less cardiac enlargement, preserved LV function, and less fibrosis after TAC when compared to mice that received the vehicle [8]. Our previous study has shown that the elevated levels of hypertrophic genes (HF indicators) such as ANP and BNP in HF murine models were markedly decreased by nitric oxide mediated production of H_2_S or H_2_S producing enzymes [9]. Similarly, in present cellular study it was observed that SG-1002 treatment of stressed HL-1 cells significantly reduced expression of HF biomarkers. Interestingly, an antioxidant property of SG-1002 was clearly observed when H_2_O_2_ induced oxidative stress in HL-1 was antagonized by SG-1002. A study showed that exogenous H_2_S not only prevented hypertrophy in neonatal rat cardiac ventricular myocytes (NRCMs), but also improved the viability of the hypertrophic cells, possibly by altering glucose metabolism [56]. 

Our results are consistent with the research on SG-1002’s impact on apoptosis, oxidative stress, and hypertrophy. Future research should examine how SG-1002 impacts other clinical HF markers, notably pro-fibrotic markers. Other studies have already found H_2_S can reduce organ fibrosis, though the mechanism of how it produces this effect is unknown [57]. In addition, other studies have found that another possible mechanism of H_2_S’s protective effect is through the mitochondria. H_2_S/CSE has been shown to decrease methylation of mitochondrial transcription factor A (TFAM) [58], which regulates mtDNA copy number and mitochondrial biogenesis [59]. H_2_S can also improve the function of mitochondria under hypoxic conditions. Under normal conditions, H_2_S can reduce ATP production by inhibiting cytochrome c oxidase [60]. However, under conditions where oxygen concentrations are low (such as in I/R injuries), CSE can be translocated to mitochondria, where it produces H_2_S, which helps to preserve the generation of ATP [61]. Furthermore, Elrod et al. found that mice subjected to MI/R injury and treated with an H_2_S donor at the time of reperfusion had preserved mitochondrial function and membrane integrity, as well as smaller infarcts, less fractional shortening, and preserved ejection fraction when compared to vehicle-treated mice [53]. With the evidence of mitochondrial involvement growing, future studies may benefit from examining the effects of SG-1002 on mitochondrial gene expression and function. 

**Figure 8:**
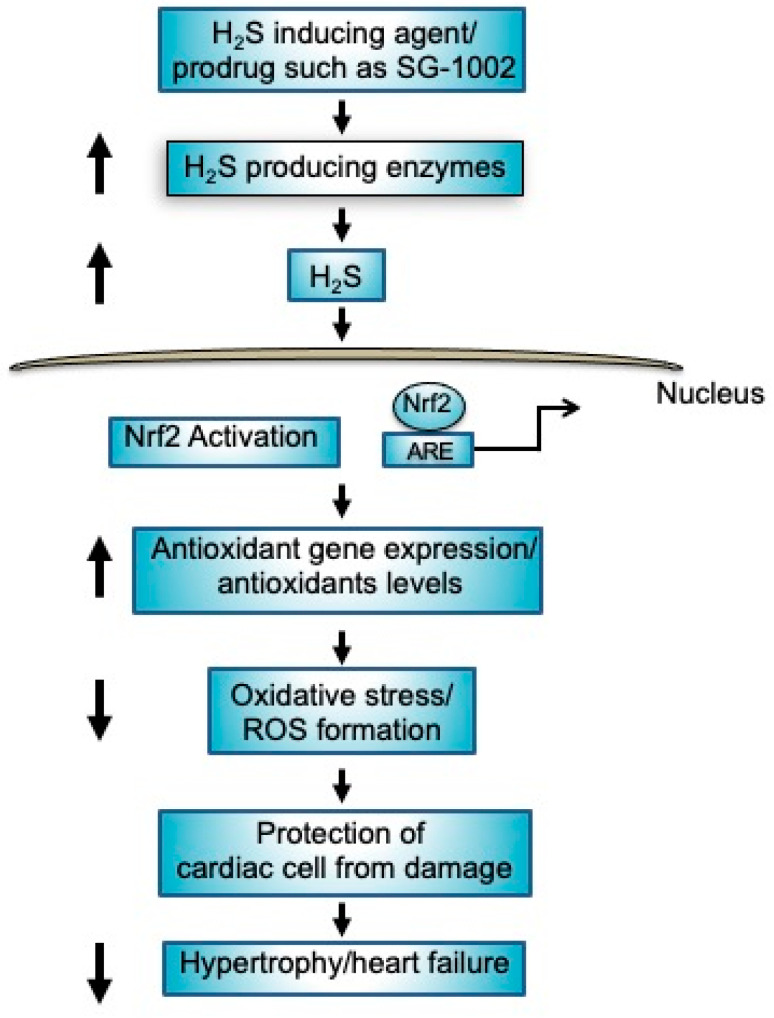
Mechanisms of protection of cardiac cells by H_2_S producing agents such as prodrug SG-1002. Treatment of SG-1002 induces H_2_S producing enzymes resulting in increased production of H_2_S. Increased levels of H_2_S activates nuclear Nrf2 as well as causing elevation of antioxidant gene expression/antioxidants levels resulting in protection of cardiac cell from damage leading to the inhibition of hypertrophy/HF.

Based on our previous findings in murine models (9), our present study may support a similar mechanism for under stressed cardiomyocytes which indicates that an increased production of H_2_S or H_2_S producing enzyme by SG-1002 may activate nuclear factor erythroid 2-related factor 2 (Nrf2), in turn causing the elevation of antioxidant gene expression/antioxidants levels and decreasing the levels of oxidative stress resulting in protection of cardiac cells from damage leading to the inhibition of myocardial hypertrophy/HF (Figure 8). 

A small (n = 18) phase 1 clinical trial found SG-1002 to be a safe and well-tolerated drug in both healthy controls and HF patients [2]. SG-1002 is emerging as a potential H_2_S pro-drug due to its safety, mode of administration, and unique ability to efficiently generate H_2_S with no byproducts in a slow and sustained, dose and enzyme independent manner. These features position SG-1002 as the H_2_S donor of choice when studying biological systems in vivo. However, its negligible solubility in water makes it a poor choice for in vitro experiments. Larger clinical studies are being designed to further test the safety and efficacy of SG-1002 for the treatment of HF/hypertrophy and CVD. Our study and others have shown that H_2_S prodrugs like SG-1002 increase the levels of H_2_S producing enzymes and antioxidants while decreasing hypertrophic gene expression and oxidative stress, making it a promising novel therapeutic drug. 

## 5. Conclusions

Data from the current study may provide a mechanism for the effects of SG-1002 on stressed cardiomyocytes. This induces the production of a H_2_S/H_2_S producing enzyme, CBS, resulting in induction of antioxidant gene expression/antioxidants levels and reduction of oxidative stress levels leading to the protection of cardiomyocytes from damage. SG-1002, via a similar mechanism, is probably involved in an in vivo system where it may protect myocardial hypertrophy/HF. SG-1002 is a leading H_2_S donor candidate due to its safety, oral activity, and unique ability to efficiently generate H_2_S with no byproducts in a slow and sustained manner, independent for both dose and enzyme. As found in a small phase 1 clinical trial, SG-1002 was considered to be a safe and well-tolerated drug in both healthy controls and HF patients. Therefore, it is necessary to study the effects of SG-1002 in cardiovascular diseases in detail through larger clinical trials.

## Data Availability

The data presented in this study are available on request from the corresponding author in accordance with the state regulations and appropriate laws.
